# Steadiness of Spinal Regions during Single-Leg Standing in Older Adults with and without Chronic Low Back Pain

**DOI:** 10.1371/journal.pone.0128318

**Published:** 2015-05-29

**Authors:** Yi-Liang Kuo, Kuo-Yuan Huang, Pei-Tzu Chiang, Pei-Yun Lee, Yi-Ju Tsai

**Affiliations:** 1 Department of Physical Therapy, Tzu Chi University, Hualien, Taiwan; 2 Department of Orthopedics, National Cheng Kung University Hospital, Tainan, Taiwan; 3 Department of Physical Therapy, College of Medicine, National Cheng Kung University, Tainan, Taiwan; The University of Queensland, AUSTRALIA

## Abstract

The aims of this study were to compare the steadiness index of spinal regions during single-leg standing in older adults with and without chronic low back pain (LBP) and to correlate measurements of steadiness index with the performance of clinical balance tests. Thirteen community-dwelling older adults (aged 55 years or above) with chronic LBP and 13 age- and gender-matched asymptomatic volunteers participated in this study. Data collection was conducted in a university research laboratory. Measurements were steadiness index of spinal regions (trunk, thoracic spine, lumbar spine, and pelvis) during single-leg standing including relative holding time (RHT) and relative standstill time (RST), and clinical balance tests (timed up and go test and 5-repetition sit to stand test). The LBP group had a statistically significantly smaller RHT than the control group, regardless of one leg stance on the painful or non-painful sides. The RSTs on the painful side leg in the LBP group were not statistically significantly different from the average RSTs of both legs in the control group; however, the RSTs on the non-painful side leg in the LBP group were statistically significantly smaller than those in the control group for the trunk, thoracic spine, and lumbar spine. No statistically significant intra-group differences were found in the RHTs and RSTs between the painful and non-painful side legs in the LBP group. Measurements of clinical balance tests also showed insignificant weak to moderate correlations with steadiness index. In conclusion, older adults with chronic LBP demonstrated decreased spinal steadiness not only in the symptomatic lumbar spine but also in the other spinal regions within the kinetic chain of the spine. When treating older adults with chronic LBP, clinicians may also need to examine their balance performance and spinal steadiness during balance challenging tests.

## Introduction

Low back pain (LBP) is one of the most prevalent musculoskeletal conditions causing physical disability and burden on individuals [1,2]. In general population samples, estimates of LBP point prevalence range from 1.0% to 58.1% and 1-year prevalence from 0.8% to 82.5% [3]. Most people who experience activity-limiting LBP tend to have recurrent episodes [3], and approximately 5–15% of people with LBP develop to a chronic condition [4,5]. Although LBP affects men and women of all ages [6], most research on LBP has been focused at the younger age or working populations [7–9]. As populations rapidly aged and older age has been identified as a prognostic factor for developing chronic back pain [10], there is a need to focus the investigation of chronic LBP in the older population.

Postural control, whether under static or dynamic conditions, is a prerequisite for independently and safely performing functional activities. People with LBP have been observed to have increased postural sway in standing [11,12]. Reduced proprioception in the spine might have contributed to this balance deficit [13]. In contrast to previous studies investigating postural control of the whole body during quiet stance, Sung *et al*. [14] focused the steadiness measurement on the lumbar spine and trunk during single-leg standing. The researchers quantified the three-dimensional segmental movement patterns in the spinal regions (steadiness index) during execution of a motor task required higher balance control. In the absence of visual feedback, participants with and without LBP showed similar abilities for maintaining steadiness in the lumbar spine. However, participants with LBP demonstrated poorer trunk steadiness than those without LBP. This unexpected finding suggests that reduced trunk steadiness was resulted from movement impairments in the other regions within the kinetic chain of the spine.

There is evidence that the lumbar spine is closely synchronized with the thoracic spine and pelvis during quiet standing and functional activities [15–17]. Gait studies in LBP patients also showed altered coordination between thorax and pelvis [18,19]. These findings indicate that thoracic spine, lumbar spine, and pelvis should be examined concurrently as linked segments. Discarding regions adjacent to the symptomatic lumbar spine may overlook important information. The research group of Sung published several studies investigating other regions of the trunk in patients with LBP during single-leg standing [20,21], however neither one study simultaneously analyzed the influence of LBP on the entire kinetic chain of the spine. Additionally, back or associated leg pain is often predominant in one side. Previous studies did not examine whether standing on the relative painful leg or non-painful leg would affect the spinal steadiness or balance performance during single-leg standing.

Besides problems in static standing balance, patients with LBP have been found to perform significantly worse than healthy controls on some physical performance tests [22,23], for example the timed up and go (TUG) test and 5-repetition sit-to-stand (STS) test. These tests are commonly used in clinical to assess dynamic balance and risk of falls [24,25]. Clinical tests measure task parameters that reflect what is required from an individual to complete the task, whereas laboratory tests measure how a task or movement is performed [26]. If the results of clinical and laboratory tests are correlated, clinical tests that are quick and easy to perform may be used to determine whether there is a need to conduct more time-consuming but informative laboratory tests. However, it is unclear whether laboratory measures of steadiness index are associated with clinical balance tests.

The majority of the above mentioned studies included LBP patients of different ages as a single group, with the mean ages typically below 60 years. Since the impact of LBP between young and older people is likely to be different, the previous findings may not be applicable to older population. Specifically for older adults, few studies have found that LBP was associated with poor balance and increased risk of falls. Compared to older adults with knee osteoarthritis, older adults with LBP had poorer performance during STS and alternative stepping, and higher occurrence of falls during one-year follow up [27,28]. Such results indicate the LBP could be a potential risk factor of falls in older population. The impact of LBP on balance and falls especially in older adults is underappreciated while knee osteoarthritis has been recognized as a major cause of falls [29,30]. Despite higher prevalence of LBP in older population, the understanding of LBP on functional balance and spinal control in older adults are still scare. From the perspective of prevention, if the balance problem of older patients with LBP could be understood, a more effective intervention may be implemented. Therefore, the purposes of this study were to compare the steadiness index of spinal regions during single-leg standing in older adults with and without chronic LBP and to correlate measurements of steadiness index with clinical balance tests. We hypothesized: (1) the steadiness index of the spinal regions would be significantly smaller in older adults with chronic LBP than those without chronic LBP; (2) the steadiness index of the spinal regions would be significantly smaller when standing on the relatively painful leg than on the non-painful leg; (3) there would be significant correlations between measurements of steadiness index with clinical balance tests.

## Methods

### Participants

Thirteen participants with LBP (9 women and 4 men) and 13 age- and gender-matched asymptomatic volunteers aged 55 years or above were recruited to participate in this study. Inclusion criteria for the LBP group were as follows: (1) the presence of non-specific LBP with a duration of at least 3 months; (2) worst pain rating on the visual analogue scale greater than 3. Participants with LBP were excluded from the study if they had any of the following conditions: a history of falls, back surgery, overt neurologic signs (severe sensory deficits or motor paralysis), specific rheumatologic diseases, spinal tumor, acute compression fracture, symptoms of fecal and urine incontinence, or any medical condition that impacts ambulation other than LBP. Participants in the control group were those with no history of LBP for a minimal one-year period and no history of falls.

### Ethics Statement

This study was approved by the Institutional Review Board of the National Cheng Kung University Hospital. Participants were recruited from the affiliated hospital of National Cheng Kung University, local communities and around the campus. Eligible participants were informed about the purpose and experimental procedure of the study, and signed a copy of the Institutional Review Board approved consent form prior to participation.

### Instrumentation

Data of 3-dimensional marker trajectories were collected using a 6-camera motion analysis system (Vicon T10, Vicon Motion Systems, 14 Minns Business Park, West Way, Oxford, OX2 0JB, UK) at a sampling rate of 100 Hz. To determine the duration of maintaining single-leg standing, force plate data were collected using one AMTI force platform (AMTI force platform model OR6-6-1000, Advanced Mechanical Technology Inc., 176 Waltham Street, Watertown, MA 02472–4800, U.S.A.) at a sampling rate of 1000 Hz.

Modified Oswestry Low Back Pain Disability Questionnaire (Chinese version) was used to rate the level of pain and disability [31]. Several basic pieces of equipment were used to test clinical balance tests, which includes a timer, a standard chair with height of 43 cm, and a cone to indicate the turning point during the TUG test.

### Procedure

Data collection was conducted at the Motion Analysis Laboratory in the university. Participants undertook the tests with bare feet. For the single-leg standing test, participants performed the test twice on both leg, and average measurement was used for data analysis. Participants in the LBP group were asked to identify which side pain is more dominant, and then performed the single-leg standing test on both relatively painful and non-painful legs in a random order. Participants in the control group also performed the test on both dominant and non-dominant legs. For each of the clinical balance tests, participants were given one practice trial and their performance on additional two trials was recorded.

#### Single-leg standing test

Ten infrared retro-reflective markers were placed over bony landmarks of participants. Specifically, the markers were attached to the following locations: bilateral acromioclavicular (AC) joints, bilateral anterior superior iliac spines (ASISs), bilateral posterior superior iliac spines (PSISs), bilateral greater trochanters, and spinous processes of the C7 and T10 vertebrae.

**Fig 1:**
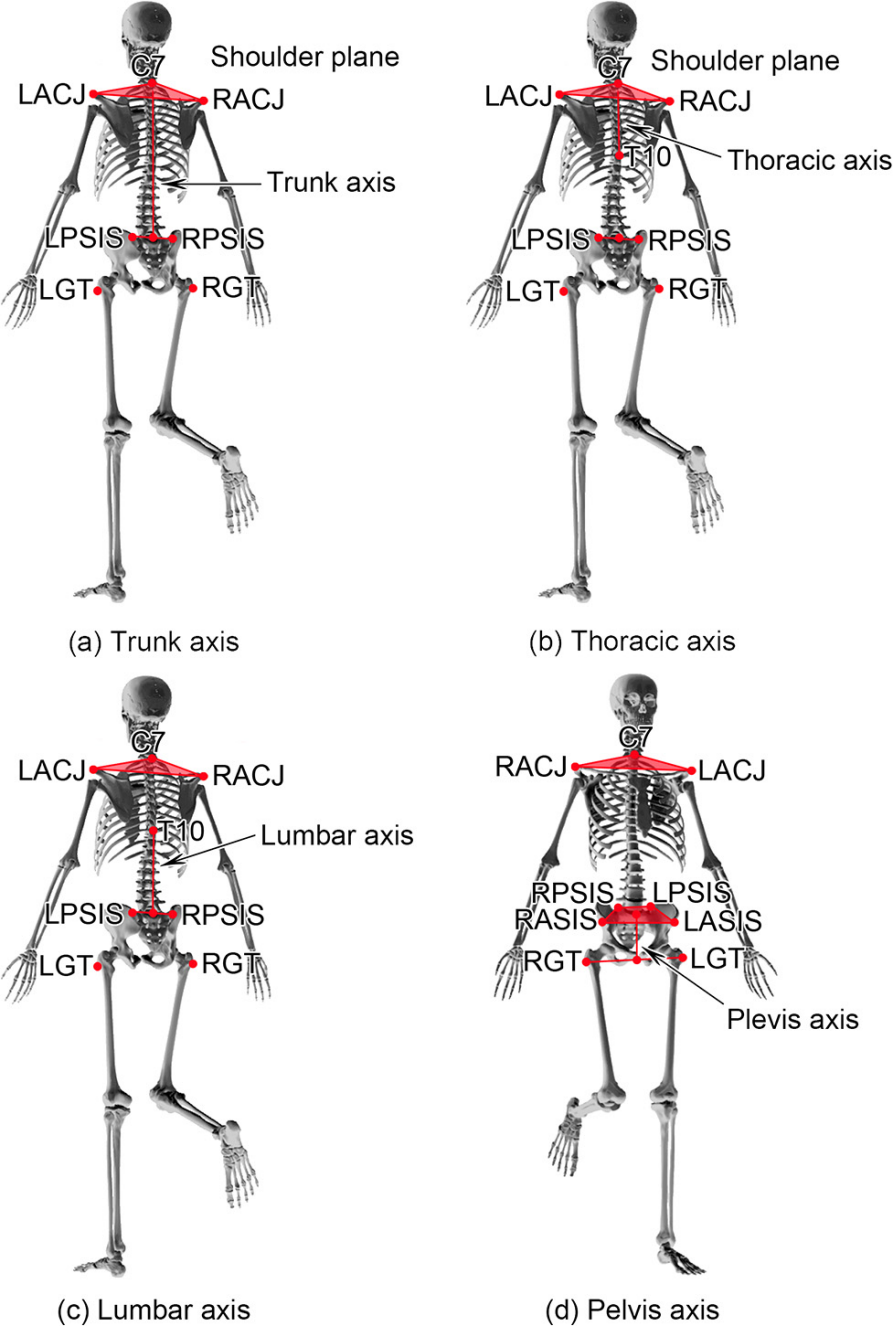
Single-leg standing test. Illustrations of a subject stands on single-leg with the contralateral knee flexed 90° for 25 seconds. Definition of each axis including trunk, thoracic spine, lumbar spine and pelvis are illustrated in (a)(b)(c)(d), respectively. RACJ and LACJ indicate right and left acromioclavicular joints, respectively. RASIS and LASIS indicated right and left anterior superior iliac spines, respectively. RPSIS and LPSIS indicated right and left posterior superior iliac spines, respectively. RGT and LGT indicated right and left greater trochanters, respectively.

After marker placement, participants were instructed to stand upright and maintain single-legged stance on the force plate for 25 seconds while flexing the other knee to 90 degrees. To avoid accidental falls during the test, participants were instructed to abduct shoulders to 45 degrees and extend both elbows (Fig 1). The holding time was measured until the flexed leg touched the ground surface.

#### Clinical balance tests

For the TUG test [32], participants were instructed to stand up from a armless, backless chair (height 43 cm), walk forward 3 meters, turn around, walk back to the chair, and sit down again. Participants were timed in seconds from the word ‘GO’ to when they are seated again correctly in the chair. The TUG test assesses dynamic balance ability and is a reliable and valid test for functional mobility and fall risk for older adults [25,32]. For the 5-repetition STS test [33], participants were instructed to rise from a standard armless, backless chair five times, as fast as possible, with arms folded closely to the trunk. Participants were also instructed to stand up fully and not to move their feet during the test. The time from the word “Go” to the moment when participants’ buttocks touched the chair after completing the 5^th^ repetition was recorded. STS has been used as a measure of functional mobility [34] and lower limb strength [35], and is included in fall risk assessment scales [33].

### Data Analysis

Marker position data during single-leg standing were exported and analyzed using Matlab program (Matlab V. 17, Mathworks, 3 Apple Hill Drive, Natick, MA 01760–2098, U.S.A.). To calculate the steadiness of each spinal region during single-leg standing, axis of each spinal region including thoracic spine, lumbar spine, pelvis, and whole trunk was determined based on a line formed from two anatomical points. The trunk axis was defined as a vertical line from the shoulder girdle plane (formed by bilateral AC joints and C7 vertebra) to the mid-point between bilateral PSISs. The thoracic spine axis was the line between the shoulder plane and T10 vertebra, and the lumbar spine axis was the vertical line between T10 vertebra and the mid-point between bilateral PSISs. The pelvic axis was defined by the line connecting the centroid of the plane formed by bilateral ASISs and PSISs to the mid-point of bilateral greater trochanters. The lumber and thoracic spine axes were parallel to the trunk axis in quiet standing position (Fig 1).

Relative holding time (RHT) and relative standstill time (RST) for the trunk, pelvis, thoracic spine, and lumbar spine were calculated respectively based on the method described by Sung *et al*. [14] RHT was calculated as a ratio between the successful holding time and the requested holding time (20 seconds after the initial 5 seconds), and RST was calculated as a ratio between the standstill time and the successful holding time. The standstill time of each spinal region was the time summation where the 3-dimensional rotation angle of the tested axis is blow the set threshold of 5 degrees. The longer standstill time indicates the spinal segment is more stable.

All data were analyzed using the SPSS 15.0 software (SPSS 15.0 software, SPSS Inc. 233 S. Wacker Drive, 11th floor, Chicago, IL 60606–6307, U.S.A.). The normality assumption for parametric statistical analysis was tested using the Shapiro-Wilk test. Data are presented as means and standard deviations (SDs). The independent t-tests and chi-square tests were used to determine whether demographic data were significantly different between the LBP and control groups. Using a two-way repeated measures analysis of variance (ANOVA) (side × region) or a two-factor ANOVA (group × region) cannot provide information regarding the difference for each spinal region. Therefore, The RHT between the painful and non-painful side legs in the LBP group was compared using a dependent t-test with statistical significance set at *p* < 0.05 (intra-group comparison). The average RHT of the control group was compared to those of the painful and non-painful side legs in the LBP group separately using independent t-tests with statistical significance set at *p* < 0.05 (inter-group comparisons). The intra-group and inter-group comparisons of RST data for each spinal region were analyzed using appropriate t-tests with Bonferroni-corrected statistical significance set at *p* < 0.0125. The association between steadiness index and clinical balance tests were determined using Spearman’s rho correlations with statistical significance set at *p* < 0.05.

## Results

Demographic data of participants in the LBP and control groups are summarized in Table 1. Both groups had similar weight, height, and body mass index (*p* > 0.05). The average Oswestry disability index for the participants in the LBP group was 27.2%, which is considered at the moderately disability level (20%–40%) [36]. There were significantly more participants in the control group who were able to hold for the whole 20 seconds on both legs during the single-leg standing test (χ^2^ = 6.5, *p* = 0.011). The control group also performed significantly better than the LBP group during the TUG (t = -2.38, 95% CI -5.4 to -0.3) and 5-repetition STS (t = -2.63, 95% CI -5.2 to -0.6) tests. Three participants in the LBP group took significantly longer to complete the clinical balance tests, exceeding the cut point of ≥ 13.5 seconds for the TUG test [25] or > 13 seconds for the 5-repetition STS test [37].

**Table 1:** Subject characteristics of the low back pain (LBP) and control group.

Characteristics	LBP (n = 13)	Control (n = 13)	*p*-value
Age (year)	60.5 ± 4.1	59.7 ± 3.0	0.558
Height (cm)	159.9 ± 10.2	161.1 ± 7.3	0.719
Weight (kg)	62.5 ± 13.5	62.5 ± 10.9	0.990
Single-leg standing*	1 (7.7%)	7 (53.8%)	0.011†
TUG (sec)	12.85 ± 4.08	9.98 ± 1.48	0.031‡
STS (sec)	11.65 ± 3.62	8.73 ± 1.70	0.015‡
Oswestry Diability Index (%)	27.2 ± 12.6	NA	-
Average pain during the past week on visual analogue scale	3.7 ± 1.4	NA	-

NOTE. Values are mean ± SD or number (percentage).

Abbreviation: TUG, timed up and go test; STS, 5-repetition sit-to-stand test; FRT, forward reach test; NA, not applicable.

*Number (percentage) of participants who were able to hold 20 seconds during single-leg standing test for both trials on both legs.

† *p* < 0.05, chi-square test.

‡ *p* < 0.05, independent t-test.

The steadiness index for all four spinal regions in the LBP and control groups are summarized in Table 2. For intra-group comparison, no significant differences were found in the RHT (t = -0.06, 95% CIs: -0.22 to 0.21) and RSTs for all spinal regions (trunk: t = 2.48, 95% CIs: 0.02 to 0.34; lumbar spine: t = 2.38, 95% CIs: 0.01 to 0.30; thoracic spine: t = 2.13, 95% CIs: -0.004 to 0.34; pelvis: t = 0.20, 95% CIs: -0.01 to 0.27).

**Table 2:** Steadiness index of spinal regions in the low back pain (LBP) and control groups.

Variables	LBP		Control	*P*-value		
	Painful side	Non-painful side		Painful vs. Non-painful	P-LBP vs. Control	NP-LBP vs. Control
RHT	0.62 ± 0.32	0.63 ± 0.25	0.87 ± 0.23	0.957	0.032*	0.017*
RST						
Trunk	0.89 ± 0.09	0.71 ± 0.25	0.96 ± 0.06	0.029	0.038	0.004†
Thoracic spine	0.87 ± 0.11	0.71 ± 0.25	0.95 ± 0.07	0.054	0.035	0.004†
Lumbar spine	0.89 ± 0.08	0.74 ± 0.25	0.96 ± 0.05	0.035	0.027	0.008†
Pelvis	0.93 ± 0.06	0.80 ± 0.23	0.96 ± 0.05	0.067	0.297	0.037

NOTE. Values are mean ± SD.

Abbreviation: LBP, low back pain; RHT, relative holding time; RST, relative standstill time; P-LBP, painful side in the LBP group; NP-LBP, non-painful side in the LBP group.

* *p* < 0.05.

† *p* < 0.0125.

For inter-group comparisons, the average successful holding time in the control group was 17.3 ± 4.5 sec, approximately 5 sec longer than the time on the painful (12.4 ± 6.3 sec) or non-painful (12.5 ± 5.0 sec) sides in the LBP group. The RHTs in the LBP group were statistically significantly smaller than the RHT in the control group, regardless of one leg stance on the painful (t = 2.28, 95% CIs: 0.02 to 0.47, *p* = 0.032) or non-painful (t = 2.561, 95% CIs: 0.05 to 0.43, *p* = 0.017) sides. The RSTs on the non-painful side in the LBP group were statistically significantly smaller than those in the control group for the trunk (t = 3.43, 95% CIs: 0.09 to 0.40), thoracic spine (t = 3.41, 95% CIs: 0.09 to 0.40) and lumbar spine (t = 3.11, 95% CIs: 0.07 to 0.37), indicating that the LBP group were less stable in these spinal regions during single-leg standing on the non-painful side leg compared to the control group. The mean RSTs on the painful side in the LBP group tended to be smaller than those in the control group; however, those differences did not reach the statistical significance level (*p* > 0.0125).

Measurements of the TUG and 5-repetition STS tests were negatively correlated with the RHT and RSTs (Table 3), which seems to indicate that the quicker participants completed the TUG and 5-repetition STS tests, the longer and more stable participants were able to maintain a single-leg standing posture. However, these weak to moderate correlations were not statistically significant (*p* > 0.05). Only 6.4–12.1% and 10.2–15.0% of the variation in the RSTs could be explained by the TUG and 5-repetition STS tests, respectively.

**Table 3:** Spearman’s rho correlation coefficients between steadiness index and performance on clinical balance tests (n = 26).

Measures	TUG test		5-repetition STS test	
	r	P-value	r	P-value
RHT	-0.262	0.196	-0.116	0.572
RST				
Trunk	-0.341	0.088	-0.387	0.051
Thoracic spine	-0.348	0.082	-0.354	0.076
Lumbar spine	-0.291	0.150	-0.353	0.077
Pelvis	-0.252	0.215	-0.320	0.111

Abbreviations: TUG test, times up and go test; 5-repetition STS test, 5-repetition sit-to-stand test; RHT, relative holding time; RST, relative standstill time.

## Discussion

Aging associated changes such as decreased muscle strength, visual impairments, and physical inactivity are known risk factors for falls among older people [38,39]. While knee osteoarthritis has been recognized as a major cause of falls, only few studies have investigated the impact LBP on balance and postural control in older adults [27,28]. The current study revealed that, in a group of older adults with no history of falls with chronic LBP had significantly smaller steadiness index of spinal regions during single-leg standing test and performed significantly worse in clinical balance tests than those without LBP. Three out of 13 participants in the LBP group, in particular, were potential fallers as indicated by the TUG and STS tests. These findings suggest that LBP may be a potential risk factor for falls in the older population. When treating older adults with LBP, clinicians may need to examine their balance performance and provide intervention to prevent potential falls.

Clinical balance tests are commonly used to identify people at risk of falling, however the use of a single test may not be adequate to assess a wide range of fall risk factors. In a recent systematic review [40], the TUG test was found to have limited ability to predict falls in community dwelling older adults, and the cut point of > 13.5 sec was more useful at ruling in rather than ruling out falls in people classified as high risk. In the current study, in addition to clinical balance tests, steadiness index of spinal regions was measured to quantify how the single-leg standing task is performed. The result of insignificant correlations between the steadiness index and clinical balance tests suggests that different aspects of balance and postural control were being measured. Therefore, while clinical balance tests are easy to perform, it might be useful to conduct laboratory measures in order to detect early signs of kinematic changes in the spinal regions during execution of a motor task required higher balance control.

The LBP group in the current study demonstrated significantly shorter holding duration and RHT than the control group regardless of standing on the painful or non-painful side legs. These results were inconsistent with the study of Sung *et al*. [14]. No significant group differences of RHTs on either side of leg were found in the study of Sung *et al*. [14] (right leg: LBP 0.89 ± 0.26 vs. control 0.96 ± 0.12, *p* = 0.17; left leg: LBP 0.88 ± 0.20 vs. control 0.96 ± 0.13, *p* = 0.07). A possible explanation for this disparity could be different characteristics of participants. The mean age of participants in the current study was approximately 20 years older than those in the study of Sung *et al*. [14] (60.5 ± 4.1 years vs. 40.7 ± 14.5 years). Older age may have exaggerated the impact of LBP on posture control, manifesting by reduced RHT during single-leg standing.

Fear-avoidance beliefs are strongly associated with disability in patients with chronic LBP [41]. Concern or fear about weight bearing to produce pain and further harm to the spine was expected to compromise steadiness of spinal regions more on the painful side leg than on the non-painful side leg during the single-leg standing test; however, the results of the intra-group comparison on the RSTs did not support our hypothesis. For participants in the LBP group, their mean RSTs on the painful side were relatively longer than those on the non-painful side, especially in the lumbar spine and trunk. Although the differences were not statistically significant, this result seems to indicate that the LBP group demonstrated a trend of better steadiness in the spinal regions when standing on the painful side leg than on the non-painful side leg.

Posture is controlled through sensory input from different body parts. Altered postural control strategies have been observed in people with LBP and in older adults [42,43]. Previous studies found that people with LBP and older adults became more sensitive to ankle muscle vibration and reweighted proprioceptive inputs from the trunk to the ankles for postural control. When standing on the painful side leg, the LBP group might have relied more on ankle proprioception as a result of pain and used more trunk stiffening strategy to maintain the single-leg stance. Thus, the LBP group had relatively longer mean RSTs on the painful side than on the non-painful side. This phenomenon was more apparent when comparing the RSTs of the painful and non-painful side legs in the LBP group separately to the average RSTs of the control group. Statistically inter-group significances were only shown on the non-painful side not on the painful side.

The result that the LBP group demonstrated a significantly smaller RST for the trunk when standing on the non-painful leg compared to the control group corresponds to the findings of previous studies [14,20]. However, spinal regions within the kinetic chain of the spine behaved differently across studies. In the study of Sung *et al*. [14], the non-significant inter-group difference of the RST for the lumbar spine implies that reduced trunk steadiness resulted from movement impairments in the other regions within the kinetic chain of the spine rather than the lumbar spine. The study of Ham *et al*. [20] supported this inference, showing that the control group had better steadiness for the upper thoracic spine than the LBP group and both groups had similar RSTs for the lumbar spine. The authors did not explain different findings between the thoracic and lumbar spine, but suggested that the LBP group might have adopted a trunk stiffening strategy [20]. In contrast to the findings of previous studies, the LBP group in the current study demonstrated reduced steadiness in both the thoracic and lumbar spine. Reduced gross muscle cross-sectional areas and muscle strength accompanied with aging [44] may have affected the ability of participants with LBP in the current study to adopt a trunk stiffening strategy. In correspondence with our interpretation, Jonsson *et al*. [45] has also suggested that the difficulties in maintaining the static single-leg standing position in older adults might depend on decreased muscle strength and endurance due to aging. Therefore, thoracic spine should not be overlooked when managing older adults with chronic LBP.

Several methodological issues warrant discussion. The research group of Sung was the first to quantify spinal steadiness during the single-leg standing test but did not provide reliability data on the test [14,20,21]. The same method was utilized in the current study without further investigation of its reliability because measurement of the RHT and RST was derived from two well-established and reliable methods. Timing the single-leg standing test has been shown to have high test-retest reliability (ICC = 0.96) in women aged 58–69 years [46] and good inter-rater reliability (Kappa = 0.88–1.0) in patients with LBP [47]. Kinematic measurement with skin marker-based motion analysis systems is commonly used in research and also has high reliability. However, reliability of the RHT and RST may require further investigation if these laboratory measures are to be used for detection of early signs of kinematic changes during execution of a motor task required higher balance control. The current study followed the method used in the study of Sung *et al*. [14] and did not analyze the initial 5 seconds of the single-leg standing test. However, Jonsson et al. suggested that the first 5 seconds are crucial when assessing balance during single-leg stance [45]. In their study, compared to young adults, older adults demonstrated a reduced decrease of force variability during the initial transition phase from standing to leg holding, resulting in difficulties in maintain steadiness during the following static phase. Analysis of the first 5 seconds of the single-leg standing test might provide additional insight in postural control of older adults with LBP. This study is the first to investigate the kinematics of the whole spine regions in older adults with LBP during a balance challenging task, and the results showed decreased spinal steadiness in certain regions. Considering movement coordination between spinal regions in future research seems sensible to better understand postural control of older adults with LBP. In addition, future research including electromyography or strength and endurance measurement of the trunk muscles would further examine the underlying mechanism for observed kinematic changes in the current study. Last, we acknowledge the relatively small sample size of the current study. All participants in the current study were community dwelling and independent with activities of daily living. The participants were generally in good health except that those in the LBP group had suffered from chronic LBP. We did not specifically question the participants their level of the usual physical activity and the presence of comorbid impairments. Those factors may affect older adults’ balance, and limit the interpretation and generalization of the results. Nevertheless, the current study could help to understand balance problem in older patients with LBP and develop a more appropriate intervention.

## Conclusions

Compared to the age- and gender-matched controls, older adults with chronic LBP had significantly smaller steadiness index of spinal regions during single-leg standing test and performed significantly worse in clinical balance tests. LBP may be a potential risk factor for falls in the older population, and affect postural control not only in the symptomatic lumbar spine but also in the other spinal regions within the kinetic chain of the spine. When treating older adults with chronic LBP, clinicians may also need to examine their balance performance and spinal steadiness during balance challenging tests.
